# Soil-Borne *Neosartorya* spp.: A Heat-Resistant Fungal Threat to Horticulture and Food Production—An Important Component of the Root-Associated Microbial Community

**DOI:** 10.3390/ijms24021543

**Published:** 2023-01-12

**Authors:** Wiktoria Maj, Giorgia Pertile, Magdalena Frąc

**Affiliations:** Institute of Agrophysics, Polish Academy of Sciences, Doświadczalna 4, 20-290 Lublin, Poland

**Keywords:** metabolic profile, mycobiome, mycotoxins, thermo-resistance

## Abstract

Soil-borne *Neosartorya* spp. are the highly resilient sexual reproductive stage (teleomorph) of *Aspergillus* spp. Fungi of this genus are relevant components of root-associated microbial community, but they can also excrete mycotoxins and exhibit great resistance to high temperatures. Their ascospores easily transfer between soil and crops; thus, *Neosartorya* poses a danger to horticulture and food production, especially to the postharvest quality of fruits and vegetables. The spores are known to cause spoilage, mainly in raw fruit produce, juices, and pulps, despite undergoing pasteurization. However, these fungi can also participate in carbon transformation and sequestration, as well as plant protection in drought conditions. Many species have been identified and included in the genus, and yet some of them create taxonomical controversy due to their high similarity. This also contributes to *Neosartorya* spp. being easily mistaken for its anamorph, resulting in uncertain data within many studies. The review discusses also the factors shaping *Neosartorya* spp.’s resistance to temperature, preservatives, chemicals, and natural plant extracts, as well as presenting novel solutions to problems created by its resilient nature.

## 1. Introduction

Conventional farming, the use of synthetic fertilizers, harsh pesticides, and commercial preservatives have proven to have a detrimental influence on the natural environment. This introduced an era of promoting sustainability and ecologically sound manufacturing methods. The European Union urges its member states to develop and practice solutions incorporating natural substances, dependences, and structures, which have an advantageous impact on the environment and human health. Numerous economical branches can “go green”; however, the paramount ones are horticulture and food production [1]. Therefore, it is crucial to identify common problems in these areas and search for optimal solutions. Predominantly reported issues include contamination and spoilage during the production and storage of food, mostly attributed to microbes. Technological advances have resolved some concerns; nonetheless, efficient methods of combating durable species are still to be established. In this review, one of the most important groups of fungi, heat-resistant fungi, is presented to explain the role of these microorganisms not only as harmful microbes for postharvest plant and fruit quality, but also as potentially beneficial organisms. *Neosartorya* spp., one of this fungal group, inhabits the rhizosphere; therefore, it has a role in shaping plant quality and sustainable horticulture.

Many countries in the European Union are large producers of strawberries. In addition, those with a share exceeding 50% of EU production are the leading producers of frozen fruits, including strawberries and concentrated juices from soft fruit [2]. These data place many European Union countries in the position of a market leader in berry production worldwide; this requires the producers and processors of fruits to monitor the quality of their raw materials, intermediates and products at every stage of postharvest production, processing and distribution, to ensure food security and enhance their market position [3]. Therefore, the issues connected with maintaining a high quality of raw materials and fruit products for both national and international markets are an important field of study, not only for the consumers and producers of food, but also for scientists. Moreover, in accordance with the latest policy initiatives and laws, such as The European Green Deal and EU Biodiversity Strategy for 2030, very important targets include reducing the use of fertilizers by 20% and making 25% of EU agriculture organic by the year 2030 [4]. Therefore, one of the aims in modern horticulture is the application of natural methods to protect crops and food, and therefore it is necessary to deepen the knowledge about the metabolic, morphological and genetic properties of *Neosartorya* fungal strains that shape their resistance to natural plant extracts, preservatives and compounds. This is important for healthy food production, given this is one of the main members of the fungal community in the plant rhizosphere, and so could be useful to developing solutions to sustainable horticulture and improving the postharvest quality of crops.

The complex morphology of ascospores and their high thermal resistance enables these fungi to survive high temperatures, including industrial pasteurization processes. *Neosartorya* spp. colonizes soil, rhizosphere and crop residues, and has the additional ability to degrade various chemical compounds, even toxic ones [5]. They overcome these barriers and are able to infect fruit; they may also pose a potential threat to thermally processed fruit products. Organisms belonging to the genus *Neosartorya*, which are present in fresh fruit, despite the lack of visible mould growth, can produce heat-resistant ascospores and, in favourable conditions, may cause the spoilage of processed fruit through rapid mycelium growth and metabolism.

Fungi have developed various adaptations that allow for them to survive when exposed to fungicides and climate change. Initially, these were mainly adaptations for protection against the harmful effects of various natural environmental stressors. However, they can also allow for them to survive in postharvest crops as dormant forms. With regards to evolution, fungi have developed additional mechanisms of response to temperature, light, humidity, oxygen, or to the presence of chemical compounds [6], which allowed for them to effectively adapt to changing environmental conditions, including resistance to high temperatures. Due to the chemical sensitivity of *Neosartorya* spp., only partial data are available. There are articles concerning the thermal death rates of ascospores of *N. fischeri* under the influence of organic acids [7] and preservatives [8]. It has been established that citric and tartaric acids destroy ascospores in fruit juices. Preservatives such as potassium sorbate and sodium benzoate are also used to control this fungus in fruit juices [9]. Delgado et al. [10] reported that hydrogen peroxide must be considered to reduce the probability of package contamination by *N. fischeri*. In recent years, the occurrence of fungal infections has been increasing everywhere; this may be explained by the changing climatic conditions and the resistance of fungi to fungicides due to their extensive use in agriculture and horticulture [11]. Bromley et al. [11] reported that the use of the most dominant class of antifungal agents, azoles, may lead to resistance in environmental fungi, which is of clinical importance. They also isolated azole-resistant examples of the *N. fischeri* species. Due to these cases, it is reasonable to carry out a study that may lead to the control of *Neosartorya* spp. by finding substances that can be used as alternative to the active compounds of plant protection agents. Therefore, this may also be achieved by testing the influence of plant extracts and food preservatives on fungal growth, and metabolic, morphological and genetic changes in these fungi. Although heat-resistant fungi have been an object of intense research, the specific nature of their metabolic profile and morphology, as well as their genome and transcriptome under the impact of chemicals, plant extracts and preservatives, is almost unknown. This review summarizes the existing knowledge concerning an important fungal group, *Neosartorya* spp., including negative and positive aspects for the environment. This review is in line with the implementation of certain research directions decided on by the European Commission, and complies with the FAO policy related to improving postharvest crops and food quality, to understand signalling mechanisms via root exudates and interactions between plant–rhizospheric microbial communities.

## 2. Characteristic of *Neosartorya* spp.

*Neosartorya* spp. are known as *Ascomycetes* and belong to the *Aspergillaceae* family. They exhibit unique heat-resistance abilities, allowing for them to withstand high temperatures. *Neosartorya* spp. fungi are considered to be a teleomorph (sexual state) of *Aspergillus* spp. and, therefore, produce ascospores. The spores are formed in groups of eight inside asci, which, in turn, are covered by an ascocarp, a large fruiting body. The ornamentation of ascospores is one of the key features enabling differentiation between *Neosartorya* species [12]. Usually, asci are differentiated into cleistothecium or gymnothecium. For example, the asci of *N. fischeri* are covered with cleistothecium, which helps them survive in a hot environment [13]. A cleistothecium is a smooth-walled, completely closed fruiting body with no designated opening. As spores are not automatically released into the environment, fungi rely on outside forces to disseminate their spores. Gymnothecium is similar to cleistothecium, with no openings and also containing asci. However, its peridial wall is a loose clump of hyphae, often entwined with coils or spines [14].

**Figure 1:**
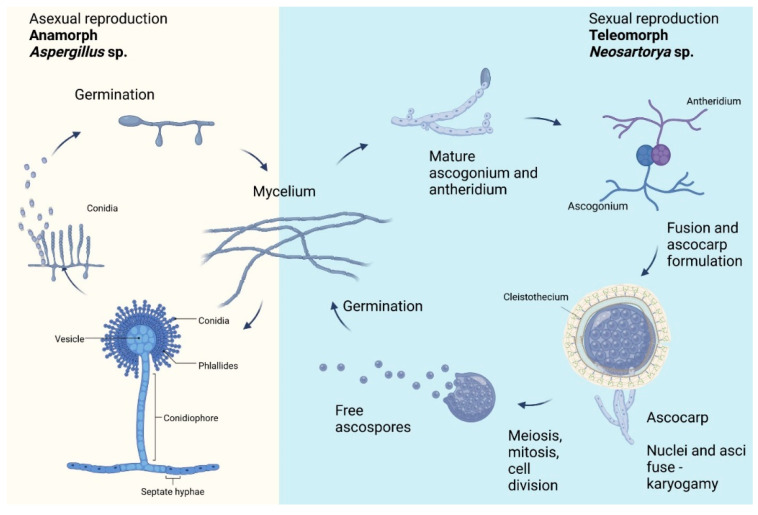
Life cycle of *Neosartorya* spp. with the stages of sexual and asexual reproduction (own elaboration by W. Maj and M. Frąc using BioRender, Toronto, ON, Canada).

The *Neosartorya* spp. life-cycle contains various phases. In general, filamentous fungi reproduce sexually and asexually. Asexual reproduction involves mitotic processes, creating conidia, whereas sexual reproduction involves meiotic processes, creating spores [15]. When a species can access both the asexual and sexual life cycles, the stages of reproduction are usually dependent on distinct environmental and nutritional circumstances. Despite the numerous benefits of sexual reproduction, over one-fifth of all fungi are only known to reproduce asexually, with no ‘teleomorph’ identified [16]. The life-cycle of *Neosartorya* spp. fungi is presented in Figure 1.

Although this approach is heavily discussed, in some cases it enables emphasis to be place on certain aspects of reproduction, e.g., the creation of ascospores. In the case of *Neosartorya*, it is the ascospores that pose the biggest obstacle, as their high thermal resistance makes them more resilient than mycelium and able to survive the high-temperature treatments used in food preservation and the postharvest storage of fruits and vegetables.

23 *Aspergillus* species enter the sexual stage and produce the *Neosartorya* teleomorph. They can all complete their sexual cycle in from approximately 2 to 3 weeks at 25 °C on a traditional mycological medium such as Malt Extract Agar (MEA) or Potato Dextrose Agar (PDA). Depending on species, strains complete the sexual cycle and generate cleistothecia with ascospores in from four weeks to six months. The cleistothecia generated by *A. fumigatus* include ascospores that are morphologically indistinguishable from those seen in other *Neosartorya* species unless studied under scanning electron microscopy (SEM). The patterns on the ascospore surface are modest yet distinct to each species [17].

**Figure 2:**
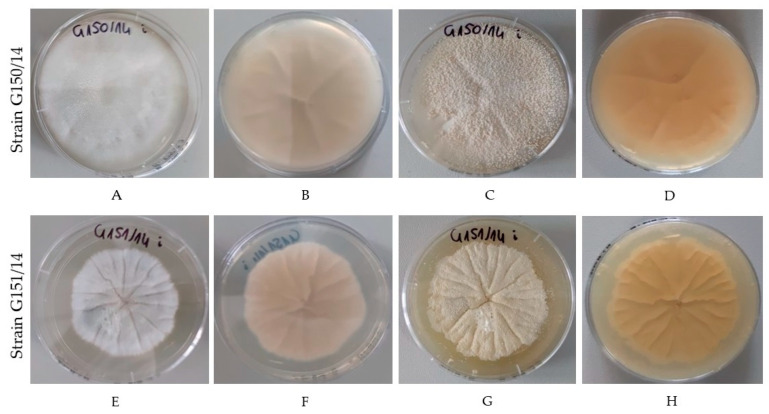
*Neosartorya* spp. growth on Potato Dextrose Agar with antibiotics (streptomycin, chlortetracycline). Strain G150/14 (**A**–**D**) after 4 days of culturing at 30 °C (**A**,**B**) and a month after (**C**,**D**). Strain G151/14 (**E**–**H**) after 4 days of culturing at 30 °C (**E**,**F**) and a month after (**G**,**H**). The number of strains: G150/14; G150/14; G151/14; G151/14 are visible on the plates presented on the photographs.

Fungal isolates identified by the β-tubulin gene sequence (Sanger sequencing, NCBI Blast) as *Neosartorya glabra* can create ascospores and cleistothecia that can be seen by the naked eye after approximately one month of culturing (Figure 2). However, they differ in the early stages of mycelium growth (A,E), producing either broad and woolly-like, floccose growth (A) or dense, velutinous colonies (E). Colonies produce larger globular ascospores that are loosely binded to mycelium (C) or finer, more powdery-like ascospores, which are better attached to mycelium (G). After a month, most ascospores can be easily detached from mycelium.

## 3. Biodiversity of *Neosartorya* spp.

To better systematize fungi, section Fumigati was created [18]. This consists of species with “uniseriate aspergilli, columnar conidial heads in shades of green and flask shaped vesicles”. The section includes 23 *Neosartorya* species. However, there are more *Neosartorya* species that are classified as doubtful and require further research, e.g., *N. australensis*, *N. ferenczii*, *N. papuaensis*, and *N. warcupii*. Usually, they differ from other taxa; in this instance based, on either their β-tubulin, calmodulin or actin gene sequences [18].

The most well-known species of the genus are *Neosartorya fischeri* and *Neosartorya pseudofischeri*, belonging to section Fumigati. They are morphologically very similar to *A. fumigatus*. The genetic diversity of *A. fumigatus* is remarkably low, especially compared to *N. fischeri* and *N. spinosa*. Moreover, *A. fumigatus* shows no geographic pattern for genetic differentiation [19]. There have been reports of *Neosartorya* spp. being mistaken for *Aspergillus* spp., proving that the differentiation between them is not obvious [20]. Despite their many close similarities, more species have been isolated and classified. For example, *Neosartorya nishimurae* and *Neosartorya otanii*, isolated from African forest soil, were characterized by their morphological differences. Both exhibited rapid growth on Czapek and Malt Extract Agars, had broad equatorial crests and lenticular ascospores. The differences between structures of cleistothecia surfaces and walls of conidia were visible. Due to their morphological affinity, some researchers question the distinctiveness of certain species. Some examples of species regarded as synonymous are presented in the table below (Table 1).

*N. spinosa*, *N. glabra*, *N. assulata*, *N. quadricincta*, *N. hiratsukae* and *N. laciniosa* are commonly isolated from fruit and soil surfaces (Table 2). They have been previously isolated from Polish soil and strawberry samples [3]. Analyses of β-tubulin gene and EcoRI RFLP patterns were most helpful in their indentification. These species, in particular, are responsible for the spoilage of food processed by heating [23].

*N. fisheri* was isolated from sunflower rhizosphere, especially after exposing plants to adverse environmental conditions, and was able to produce inulinase, which is important for the food industry as an alternative for the production of fructose syrups [24]. *N. hiratsukae* has also been reported indoors, in the air, on drywall in an Italian hospital. The small white colonies were hardly visible on white walls, so the spores could easily spread. Their presence in the environment caused a health risk, as they could lead to aspergillosis and other infectious diseases [25].

**Table 2:** Common *Neosartorya* spp. species and their properties.

Name	Telomorph	Relation to Other Species	Key Characteristics	Type of Growth	Reference
*N. spinosa*	*Aspergillus fischeri* var. *spinosus*	Has identical partial beta-tubulin and calmodulin gene sequences to *N. botucatensis* and *N. paulistensis*	Rough ascospores	On MEA: broad growth in pale yellow or yellowish white colour; thin layer of mycelium and abundant, granular cleistothecia	[12,26,27]
*N. laciniosa*	*Aspergillus laciniosus*	Closely related to *N. coreana*	Microtuberculate ascospores with two bent crests and two distinct equatorial rings of small projections	On MAA: beige with light yellow ascospores; on CYA: light yellow and white growth	[12,22,26]
*N. glabra*	*Aspergillus fischeri* var. *Glaber*	Extrolites typical for *N. fennelliae*, but is more closely related to *N. denticulate*, despite having divergent ornamentations of ascospores	Confirmed to be a disease agent; homothallic species	Yellow–white to pale yellow cleistothecia, smooth ascospores	[12,22,28]
*N. assulata*	*Aspergillus assulatus*	Closely related to *A. waksmanii* with only 2% bp difference in the act1 locus	Common extrolites produced by its colonies are indole alkaloids and apolar metabolites	Snow-white growth on MEA medium	[22,29]

## 4. Two Sides of the Same Coin

The *Neosartorya* genus consists of extraordinarily heat-resistant fungi, which are immune to high temperatures and, consecutively, food preservation techniques, utilizing them. Acidic crops and produce that cannot undergo thermal conditions higher than 60–65 °C are especially vulnerable. In these circumstances, *Neosartorya* spp. can sporulate with great efficiency. Certain species require high temperatures to sporulate, meaning that thermal processing may result in the sudden appearance of new fungal growth [30]. This leads to the secretion of mycotoxins, e.g., aflatoxins, fumitremorgins and gliotoxin, which can pose a threat to both plant and human livelihood [31]. Fornal et al. [32] developed a method that enables the fast and easy quantification of mycotoxins typical for *Neosartorya* spp. isolates, including fumitremorgin C and verruculogen in strawberries, strawberry juice, potato dextrose broth and soil. As *Neosartorya* spp. is present in the soil, the transference and subsequent contamination of plants that come into contact with the ground is effortless. Contaminated plants pass the pollutant to crops, which, in turn, are harvested, processed, and eaten by humans. Without effectively breaking the life-cycle of *Neosartorya*, its extrolites can be transmitted to the food chain, posing a threat to peoples’ well-being. As a precaution, new laws have been established regarding the quality of produce. In accordance with these principles, food that is not of satisfactory purity is not utilized [33,34].

It is important to discriminate between the species of *Neosartorya* and *A. fumigatus* in the food industry. Even subtle differences in genotype may lead to different reactions to chemical agents and treatment methods. Moreover, *Aspergillus* fumigatus has never been reported as a spoilage agent in heat-processed food products, meaning that its detection may not foreshadow future concerns [23]. Furthermore, although *N. fischeri* and *A. fumigatus* are phylogenetically close, they have different patterns of carbon sources’ metabolism [35].

*Neosartorya* spp. is also an infectious agent. Recent studies show that, due to the misidentification of fungi, *Neosartorya* genus may be as infectious as *Aspergillus* spp. Aspergillosis, an illness caused by *Aspergillus*, is a major cause of human morbidity and mortality, with over 200,000 life-threatening infections each year worldwide [36]. There had been reports of *Neosartorya hiratsukae* causing the same disease. It is often wrongly identified as *A. fumigatus* due to its close morphological similarity. It also cannot be differentiated by the popular matrix-assisted laser desorption/ionization time-of-flight mass spectrometry (MALDI-TOF-MS) that is used in many medical analyses [37].

A comparable situation arose with *Neosartorya udagawae*, which is also often mistaken for *A. fumigatus*. In 2014, it was reported to cause acute respiratory distress syndrome (ARDS) in a 43-year-old woman [38]. It was distinguished by sequencing ITS, calmodulin and β-tubulin genes. Due to this fact, further research about identification tactics is needed for therapeutics to be more successful, because of differences in the susceptibility to antifungal drugs. *Neosartorya* has also been proven to cause other diseases, such as endocarditis (*N. fischeri*) [39] or dermatitis (*N. hiratsukae*) [40].

*Neosartorya* spp. is notoriously known for producing durable, toxic metabolites. *N. fischeri* can synthetize acid protease and glycoside hydrolase (GH) 27, which, if left uncontrolled, may be potentially dangerous to wood, fiber and plants [41,42], but also very useful for industry. The *Neosartorya* spp. strain BL4 is known to biodegrade petroleum hydrocarbons. This might be useful when developing bioremediation techniques; however, it also proves how unsusceptible these fungi really are [43]. *A. flavus* and *A. parasiticus* produce aflatoxins that are toxic to the liver and are carcinogenic: the consumption of contaminated groundnuts has been linked with hepatic carcinoma in the populations of Africa and Asia [44]. Furthermore, *Neosartorya pseudofischeri* produces dangeurously cytotoxic metabolites, which are proven to cause harm to Sf9 cells from *S. frugiperda* [45]. This, in combination with enzymes, mycotoxins and other extrolites, suggests that *Neosartorya* spp. can be seen as a health hazard, and a possible threat to food production and the economy.

**Figure 3:**
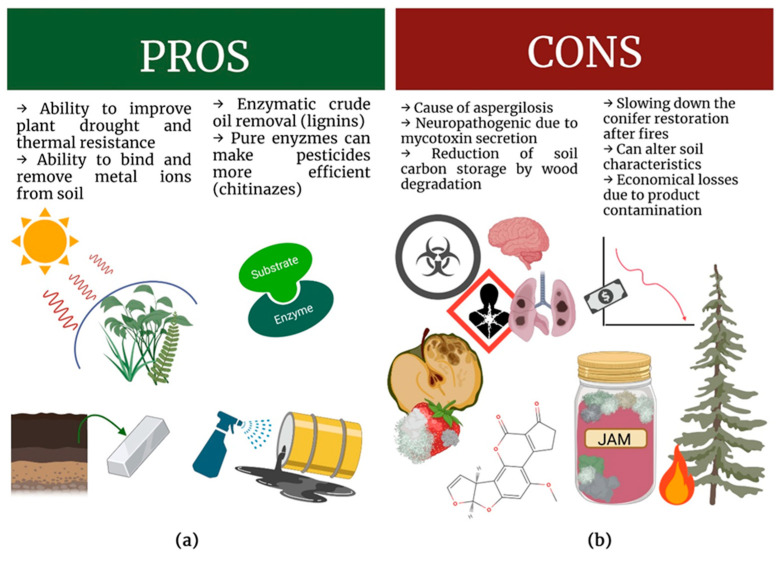
Positive and negative aspects of *Neosartorya* spp. (**a**) Positive aspects, (**b**) negative aspects; Own elaboration by W. Maj and M. Frąc using BioRender, Toronto, Canada. Aflatoxin B1 structure molview.org (accessed on 12 October 2022).

However, in a controlled environment, *Neosartorya* spp. may prove useful in agriculture, horticulture and medicine (Figure 3). It exhibits antibacterial and antifungal properties [46,47] and can be used to produce nanoparticles to control brown spot in rice [48]. New research suggests that *Neosartorya* spp. could be used to develop novel cancer treatments [49,50]. It can also help in the production of medicine for diabetes [51].

*Neosartorya* spp., as with most filamentous and heat-resistant moulds, can be seen as either a threat or a tool (Figure 3). On the one hand, molecular biology can utilize *Neosartorya*-derived proteins or use it as a binding factor in environment protection. On the other hand, its potential as a general health and economic hazard makes it an unwanted contaminant.

## 5. Heat Response in *Neosartorya* spp. Fungi

Heat resistance, or thermotolerance, is facilitated by a range of factors and processes. These include the presence of heat-shock proteins, molecular chaperones, chaperonins, protective substances, the innate properties of proteins that contribute to making them thermostable, cell-wall stoichiometry and architecture, the formation of multicellular structures, and the development of spores [52]. In general, there are two primal types of thermotolerance: basal and acquired. The first describes an organisms’ ability to survive high temperatures without prior acclimation. The latter refers to thermotolerance acquired during prior exposure to mild temperatures, which are not harmful to the organism [53]. To more precisely describe the types of heat responses, organisms can be further divided into different categories. We can observe a variety of lifeforms, such as: (a) cryophiles (psychrophiles), which are capable of life functions at −20 °C [54]; (b) sychrotolerants (psychrotrophiles), which are capable of growth at low temperatures but possess optimal and maximal growth temperatures at the 15–20 °C range [55]; (c) mesophiles, which are capable of growth at moderate temperatures between 20 °C and 45 °C, with an optimum growth temperature in the range of 30–39 °C [56]; (d) thermoduric organisms, which are capable of growth in the mesophilic temperature range (15–37 °C), yet retain the ability to grow at refrigeration temperatures [57]; (e) thermophile, which possess the ability to resist elevated temperatures, enabling them to colonize new environmental hyperthermic niches. The developement of thermophilia was probably based on pre-existing molecular blocks, as it shares many mechanisms with the heat shock (HS) response [58]. Both thermoduric and thermophilic microorganisms can withstand pasteurization, especially as spores [59].

In dimorphic fungi (e.g., *H. capsulatum*), morphology and temperature are linked with each other. This connection enables a conversion from filamentous to yeast at an elevated temperature and vice versa [60]. Moreover, heat resistance can differ between the strains of a species [61].

The vast majority of yeasts and moulds are resistant to heat in the same way as mesophilic vegetative bacteria. The heat resistance of sexual spores and asexual conidia is not greater than that of vegetative cells. However, ascospores of some moulds, such as *Byssochlamys*, *Neosartorya*, and *Talaromyces* species, have a relatively high heat resistance, with a 7–22-min D value at 88 °C, and may survive 30 min of heat treatment at 90 °C, causing microbial spoilage in processed fruit drinks and canned fruits [61].

### 5.1. Impact of the Environment

Alvarenga et al. [62] extracted data from publications between 1969 and 2017 about thermal resistance parameters and their effects on heat-resistant fungi belonging to the *Neosartorya* genus. Data included a comparison of the effects of decimal reduction time (D), inactivation method, temperature of inactivation, pH, °Brix, maturity of spores, and kind of medium (model, juice, concentrates). Each of these parameters can impact fungal heat resistance [62]. These results also indicated that, for *Neosartorya* spp., the estimate for pooled D* values (D at 90 °C, pH 3.5 and 12° Brix) was: 5.35 min; 95% CI: 4.10–7.08 min. Moreover, increasing the content of soluble solids in concentrates tends to cause a smaller decrease in the heat resistance of *Neosartorya* and ascospores appear to be more thermal-sensitive to a decrease in medium pH [62].

Brix can be defined as a measurement of the dissolved sugar-to-water mass ratio. Thus, it can be connected with the dilution of medium. Naturally, freshly squeezed vegetable juice has a lower Brix value, between 5 to 12. Concentrates, caused by the thermal evaporation of water, have higher Brix values, between 25 and 60 [63]. Results obtained by Lane Paixão dos Santos et al. [64] confirmed that increasing °Brix lowers the livelihood chances of *Neosartorya*. Within tested species, *N. udagawae* was the most resilient and possessed the ability to grow at the highest evaluated °Brix (59°/aw = 0.86) [65]. Similarly to sugars, the concentration of other substances can have an effect on heat resistance. For example, NaCl, used to decrease the water activity (aw), causes an increase in the heat resistance of some microorganisms. NaCl, used in up to 10%, increases the thermotolerance of Salmonella and acts as a heat-protectant for *L. monocytogenes* [66]. To summarize, a reduction in water activity considerably boosts heat resistance. This is a common issue with foods abundant in sugar, proteins, or fat. However, acidic pH substantially lowers heat resistance. The pH of 4.5 marks a crossing point, as goods with a pH of less than 4.5 can be pasteurized at 100 °C or lower, but foods with pH greater than 4.5 must be sterilized at temperatures higher than 100 °C. The primary reason for this is a microbe, *C. botulinum*, which cannot grow or create toxins at pH 4.5, and any of its spores that survive heat treatment cannot germinate properly. The interplay between heat and other variables can be advantageously used in food production [61].

### 5.2. Heat Shock Proteins—HSPs

Heat shock proteins (HSPs) are part of the protective mechanism of cells in case of stress. They have a biological function and are involved in transcription, translation, protein folding and posttranslational modifications. HSPs maintain the quality of proteins. In fungi, HSPs are triggered by either specific (temperature shock) or general (pH, starvation, other stress factors) mechanisms [66].

The most common HSPs in fungi are: Hsp90, Hsp70, and Hsp20–40. They play a crucial role in changes in the morphology, adaptation procurement and shaping of anti-fungal resistance [60]. Hsp90 has been studied in *A. fumigatus* by Lamoth et al. [67]. Hsp90 plays a key role in morphogenesis, helps with transcriptional regulation and controls conidation [67,68]. According to the UniProt Database, the repression of its gene showed decreased spore viability, decreased hyphal growth and defects in germination and conidiation. Moreover, Hsp90 is distributed throughout cytosol and moves to specific organs during stress. Hsp90 and Hsp70 were found in *N. fumigata*. Alone or combined, they play a major role in morphogenesis and dimorphism. *N. fumigata* also produces Hsp104, Hsp70, and Hsp40, which play a role in replication [60]. HSPs 70 exhibit ATPase activity and disaggregate denatured proteins, which, in turn, helps with proper chain folding de novo [69].

### 5.3. Trehalose and Mannitol

The processes utilized by thermophiles to create resistance to increased temperatures are similar to those used by mesophilic fungi in the heat shock response. For example, under heat shock conditions, mesophilic fungi increase the amount of trehalose up to 6–8% of dry weight, which is similar to the values in the thermophilic fungus *Myceliophthora thermophila* (up to 3.5%) under optimum temperature conditions. Mesophilic fungi can acquire thermotolerance by HSP synthesis, trehalose accumulation, changes in the state of water in cell compartments, and membrane composition [58].

Trehalose is the most widespread naturally occurring disaccharide. In some fungi, only acid (unregulated) trehalase has been found (e.g., in *Aspergillus oryzae*) [69]. Wyatt et al. [70] identified and characterized a series of trehalose-containing oligosaccharides responsible for the unique preservation properties of *Neosartorya fischeri* ascospores. In vivo, they acted as a shield for cytosolic biomolecules [71].

The activity of trehalose gene NTH1 is multiplied by ten during the heat shock reaction. During the conidia germination of *A. nidulans*, neutral trehalase is responsible for trehalose mobilization and glycerol build-up. According to tests on *Aspergillus oryzae*, mannitol substantially inhibits acid trehalase from conidia cell walls. Trehalose accumulates during the idiophase, when growth activities are inhibited. Having reached its peak in resting form, it is known as the dormancy sugar. In the early stages of *A. niger* conidia germination, trehalose levels are found to be significantly lower. *A. niger* has proven to be capable of modifying its trehalose and glycerol levels in conidia, indicating the existence of adaptation mechanisms comparable to those seen in vegetative cells. The antioxidant defense process under heat shock comprises not only of the activation of desaturase activity but also the stimulation of trehalose production [69].

Increasing the heat resistance capacity has as much to do with trehalose as it has to do with mannitol. These substances and their relationship are involved in securing the livelihood of cells during oxidative stress. The mannitol and trehalose metabolism cycles are closely connected. Lowering the concentration of mannitol causes an increase in the amount of trehalose and trehalose-based oligosaccharides present [70].

### 5.4. Other Metabolites

*Neosartorya* spp. produce many resilient metabolites, which can upkeep the metabolism even under high temperatures. For example, a purified exo-polygalacturonase (EplNg) of *Neosartorya glabra* was effectively identified. The enzyme was active from 30 to 90 °C, with the highest activity at 65 °C and pH 5.0 [27]. Another highly active thermophilic enzyme has been discovered in *N. fischeri* P1. It has been dubbed the soybean isoflavone glycoside-degrading-glucosidase of GH3. The enzyme exhibited a greater optimal temperature and specific activity than any other known fungal homologue, was stable across a wider pH and temperature range, and was resistant to the majority of tested compounds. It had broad substrate specificity, including glucosidase, cellobiase, xylanase, and glucanase activity [72]. *Neosartorya fischeri* M-1 developed a thermophilic glucoamylase that was most active at temperatures ranging from 55 to 60 °C, and had the maximum activity at pH levels ranging from 4.0 to 4.4. Producing enzymes that are stable in hot conditions are definitely beneficial for the heat-resistance shaping of fungi. These enzymes can act as tools, enabling survival and growth in a heated environment [73].

## 6. Interactions between *Neosartorya* and Plants

Representatives of *Neosartorya* genus are a widely detected fungal group, mostly inhabiting soil. Therefore, it is easily transmittable to plants, impacting their postharvest quality. There are numerous studies in which either *Aspergillus* or *Neosartorya* phases were detected on plants and fruit, e.g., strawberry [30,32,35], coffee plants [74], apples [75], grapes [76]. The spectrum of fungus–plant interaction is broad, beginning with the roots and ending with the very top aboveground plant organs. The presence of *Neosartorya* on plant roots can either be characterized as opportunistic for the fungus or mutually beneficial for both parties. In the first instance, the fungus may be attracted to damaged or diseased roots due to its saprophytic nature. It then accelerates rot and spoils the plant further. Such a situation has been described by [77], where pineapple plants suffering from the red leaf disease had reduced root systems and tested positively for *Neosartorya fischeri*, which does not cause said disease. In this case, the mycelium did not spread to aboveground organs. Often, *Neosartorya* can create a symbiosis with its host, acting as a natural antimicrobial agent or promoting plant growth by enzyme secretion. *Neosartorya fischeri* has been reported to inhabit a traditional medicinal herb *Macleaya cordata*, mainly distributed in China [78]. Interestingly, it acted as an endophytic organism, providing its antibacterial properties to both the plant and, later, to people consuming the plant for its medicinal value. The antimicrobial activity of *N. fischeri* in this study has been proven against eight bacteria: *Agrobacterium tumefaciens*, *Bacillus subtilis*, *Staphylococcus aureus*, *Staphylococcus haemolyticus*, *Salmonella typhimurium* and *Xanthomonas vesicatoria*. Hamayun et al. [79] reported gibberellins’ production and the growth-promoting capacity of another endophytic *Neosartorya* strain (CC-8), which was isolated from the roots of Chinese cabbage (*Brassica rapa*). The fungus cultures significantly promoted plant length and biomass gain.

**Figure 4:**
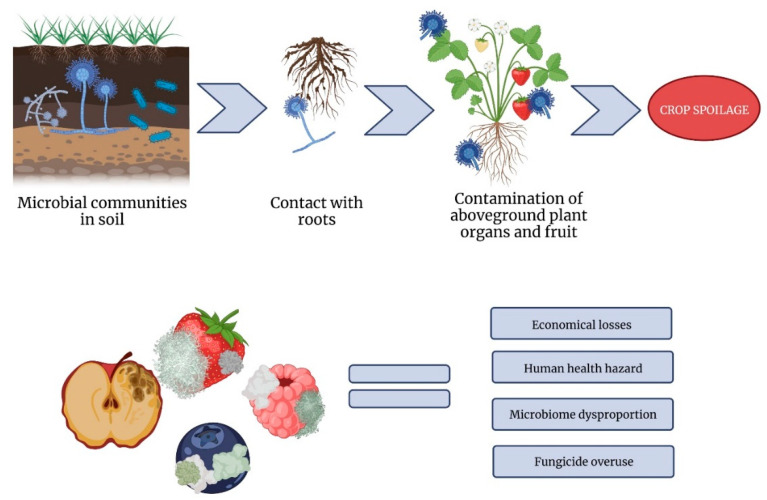
Ways of causing postharvest food spoilage by *Neosartorya* spp. (own elaboration by W. Maj and M. Frąc using BioRender, Toronto, ON, Canada).

*Neosartorya* spp. can migrate to plant organs other than the roots (Figure 4). It has been reported on leaves of kale, where, in consortium with *Talaromyces*, it was showed to control leaf spot in kale, caused by *Alternaria brassicicola* [80]. *N. spinosa* yielded the best results against this pathogen amongst all tested strains (others were *N. hiratsukae*, *N. pseudofischeri*, *N. aureola*, *N. spinosa*, *N. fennelliae*, *Neosartorya* sp., *T. trachyspermus*, *T. muroii*). Genus *Neosartorya* is also one of the five main groups of fungi present on fresh common reed (*P. australis*) leaves, where they exhibit co-occurrences with other members of *Ascomycota* and *Basidiomycota* [81]. They also act as saprotrophs, transforming dead plant matter into compost.

## 7. Fruit and Vegetable Production in the European Union

### 7.1. Organic Crop Production

Organic farming can be seen as a viable alternative to high-input horticultural systems relying on synthetic fertilizers, fungicides, and insecticides. It is built on the premise that the soil is a living system, closely intertwined with fauna and flora. It considers the microbiome and its interactions with the soil–plant system. Laws define the word “organic” mostly in terms of ‘natural’ vs. ‘synthetic’ inputs [82]. The most common practices used in sustainable horticulture are crop rotation, utilizing animal manure, and biological pest management [83]. There are many ways of delivering additional nutrients to the soil, e.g., mineral fertilization (increases the ground’s mineral content) and organic manuring (upkeeping the soil’s biological fertility) [84].

Organic crops possess more value than regular fruits and vegetables. They are often richer in nutrients [85] and contain fewer heavy metals [86]. Due to the current holistic view of ecological behaviours, the volume of organic crops is steadily growing. Organic horticulture promotes not only the production of food, but also the production of fibre and timber [83]. The branding of produce as “organic” is heavily controlled. Organic farming methods are sustainable, have a minimal environmental effect and may be viewed as a means of cleaning up and rehabilitating deteriorated agricultural land [82].

Horticultural goods are an important aspect of the European Unions’ regional and cultural character. According to Eurostat data from 2019, Poland was one of the European producers with the highest yield of organic crops. The most important producers in the EU were France, Spain and Italy. According to Eurostat in these countries the total amount of harvested crops in the EU in 2019 included grain (299.3 mln t), vegetables (61.5 mln t) and fruit, berries, nuts (25.2 mln t), respectively. Furthermore, in this year, organic crop farming accounted for 8.5% of the EU’s total utilised agricultural area, with 13.8 million hectares available for growing organic crops.

### 7.2. Poland as a Leader in Fruit Production in the European Union

**Figure 5:**
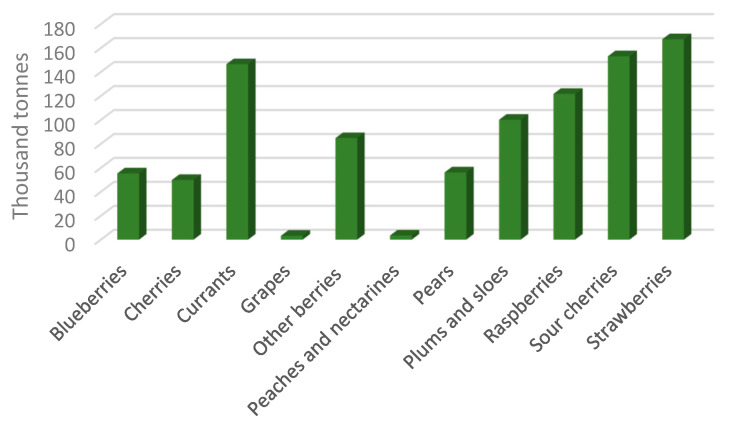
Polish fruit production in 2020 (data from fao.org) (accessed on 12 October 2022).

**Figure 6:**
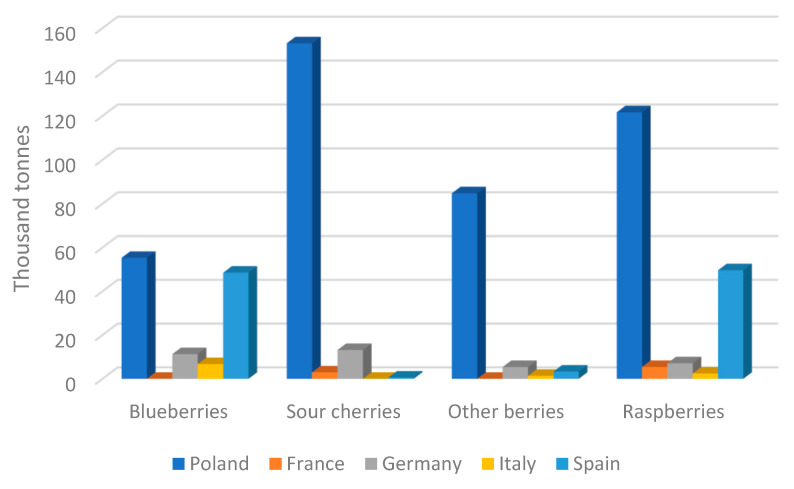
European fruit production in 2020 (data from fao.org) (accessed on 12 October 2022).

The food industry in Poland was among those sectors that saw significant upheavals and rebounded quickly following the country’s political revolution in the 1980s. As a result, the industry became an important part of the economy, influencing economic growth. Poland has evolved into a sophisticated and innovative food manufacturer in Europe as a result of technical and organizational advancements. This is proven by the increase in food exports. Another significant aspect that aided the growth of this business was Poland’s entrance to the European Union and the resulting prospects for the greater exploration of other markets. Polish food makers were eligible for various forms of grants and subsidies as a result of their EU membership. The standards for food production in Poland are mostly established by European Union legislation. Compliance with EU rules and regulations is especially critical for food exporters, since about 80% of Polish food exports are destined for EU markets. In addition to strict norms and novel pro-ecological legislation, consumers’ interest in food produced by industrial methods has rapidly declined. Chemical-plant-manufactured goods or genetic alterations being utilized in production make customers hesitant to purchase. Thus, it is important to implement new, ecological technologies in food production and preservation [87]. Ecologically sound alternatives should not omit the threat posed by thermoresistant organisms. As reported by Eurostat, statistically, in 2016, Poland harvested over 1 of every 4 apples produced in the EU. Poland was also the main EU-producer of cherries and the second most important producer of strawberries, right after Spain. In general, according to the National Centre of Agricultural Support (KOWR) Poland is a major producer of strawberries, gooseberries, and chokeberries. The fruit harvest from orchards was estimated to be 4.5 million tonnes in 2018. Based on data reported by FAO, Figure 5 and Figure 6 present fruit production in 2020 in Poland and Europe, respectively.

The production of cherries and apples accounted for a sizable portion of this total. In 2017, Spain represented the most noteworthy extent (40.1%) of the region inside the EU in terms of organic food production, due to high yield of nuts and citrus products. Italy represented the following most noteworthy country (17.5%), followed by Poland (9.6%). According to KOWR, the current estimate is that 350,000 tonnes of apple juice concentrate are produced, representing a 29% growth over the years 2014–2017. While these numbers seem optimistic at first, it is important to remember that a high yield does not always equal rapid income. After harvest, fruit is still susceptible to rot and pests, which can generate economic losses.

### 7.3. European Union Policy Framework

The European Union is currently focused on supporting ecological solutions in many sectors, including horticulture. Legal documents backing the EU’s support for sustainable farming include, e.g., regulation no. 1308/2013, focused on the common organisation of the markets in horticultural products, directive 2009/128/E, touching on the subject of pesticide usage, and Water Framework Directive 2000/60/EC (WFD), directly impacting which substances may be used in plant protection in relation to water quality and purity. The “umbrella” directive that summarizes the EU’s goals is the common agricultural policy (CAP). The CAP aims to combat climate change, conserve natural resources, and promote variety in the EU. It supports sustainable agriculture and horticulture by recommending reduced pesticide and fertilizer usage and supporting organic farming. The CAP greatly contributes to the decrease in the overall impacts of food manufacturing. It is important to uphold EU standards regarding horticultural development, and especially important to follow the trends of sustainability and creating positive environmental impact. Inventing novel, greener alternatives to commonly used preservation methods of obtained crops can further help to implement these policies in real life [88,89,90].

### 7.4. Common Problems in Fruit Production

Fruit production faces many difficult challenges. The issues can be divided into the following: (a) biological, including vulnerability to pest, diseases, microbes, and postharvest losses due to these factors, and (b) economical, including poor pricing and low fertilizer use, the unavailability of horticultural credit, land tenure insecurity, the slow development of horticultural research, infrastructure, the productivity of labour, and consumer expectations [91,92,93].

Looking at the presented data, it is crucial to develop new technologies for food protection, harvest and processing. This can be achieved with further research on pathogens’ heat resistance, methods of detection and natural food preservatives. As explained before, fruit production plays a major role in European and Poland’s economy, and any losses in this sector could be grossly disadvantageous.

### 7.5. Methods of Postharvest Food Preservation

The destruction caused by postharvest microbiological food contaminants, including heat-resistant fungi and diseases, amounts to a 20–25% yield reduction, depending on the country [94]. Therefore, the methods, strategies and ways of postharvest food protection are very important. The current postharvest strategies of microbiological contaminants’ mitigation heavily rely on chemicals, which pose a threat to the environment and human health. Several techniques can be used to protect against deteriorating factors, e.g., freezing or chilling, pasteurization, canning, or dehydrating [30,61]. Pasteurization and canning usually have little effect on ascospores, which later sprout into fungi and create mycotoxins that contaminate the produce. Mould ruins the product by developing colonies on the surface, floating mycelia, or clarifying the material. As a result of their microaerophile nature, they destroy fruit juices even when stored under low-oxygen conditions. *N. fischeri* is frequently responsible for the deterioration in apple juice, strawberry pulp, and passion fruit juice. As previously mentioned, harvest losses in these commodities can cause serious economic problems in Poland, which is a key producer of apples, cherries, and strawberries in the EU.

In accordance with European Union laws and suggestions touching on the subject of sustainable horticulture and food production, the use of heat or natural preservatives to protect the produce is highly recommended. Plant extracts may present a valid means of protection against fungi. It has been confirmed that *Calendula arvensis* hydrosol extracts possess antifungal properties, mainly against *Penicillium expansum* and *Aspergillus niger*. However, it is unclear whether essential oils or hydrosols have the best antifungal properties. This pertains to the concentration of substances, but also the type of plant material. Extracts diluted in water are generally seen as milder and safer to use in food production than highly concentrated essential oils, which mainly dominate the field of cosmetics [95]. Despite marigold being more active as a hydrosol, plants such as mint [96], thyme and lavender [97] express wider antifungal properties in the form of essential oils [98].

Tipping the heat-resistance temperature point may be another green alternative to chemical protection. For example, raising the pasteurization temperature to 95 °C for at least 45 s successfully lowers the risk of purees being spoiled by A. fischeri. Furthermore, it increases the microbiological stability of such purees [30].

Subsequently, simply editing the environment of fungal growth may be enough to stop its growth altogether. Previously mentioned variables, such as °Brix/acidity ratio [99], pH [100], °Brix [101], alternating temperatures, and the concentration of soluble dry matter can all be altered to elicit a reaction from fungi [102]. There are also studies that show the prospect of using the UV-C light as a form of non-thermal food processing. This is considered to cause few quality alterations while reducing microbial burden [103]. During transport, it has been proven that using a coating with lipopeptides and nisin on cardboard boxes can diminish the duration of *Neosartorya hiratsukae* [104], which might prove advantageous during the shipment and delivery phase of production. Coating composites are used to improve the postharvest quality of fruits [105,106,107], and can be effective in the control of fungi, including the genus of *Neosartorya* [108].

## 8. Conclusions and Future Directions

*Neosartorya* spp. fungi are riveting and extraordinary organisms, which require more focus and research. Being easily mistaken for *Aspergillus* has masked their actual contribution to food spoilage and effects on human health. They simultaneously pose dangers to crops and offer novel perspectives in medicine. In the future, more studies focused on *Neosartorya* are needed to estimate its inactivation parameters in food industry, and deepen the knowledge of its infectious properties and possible medicinal uses.

Moreover, future research directions should focus on heat-resistant fungal threats to horticulture and postharvest food security to develop new approaches to ensuring a robust global food supply chain. An important issue of future research is to consider and obtain insight into the plant mycobiome as important player in enhancing resistance to microbiological food contaminants and pathogens. Another challenge is to determine how to protect crops from postharvest fungal damage, especially from the *Neosartorya* genus. The best strategy at present may be to focus on studying natural fungicides and substances, such as microbial-based solutions, plant extracts, and essential oils, which have little to no effect on the environment and do not lead to the decay of postharvest horticultural products and food, but do affect thermoresistant moulds such as *Neosartorya*.

Finally, effort should be taken to find connections between heat-resistant fungi and climate change, including responses to the question of how new and existing fungi can be identified, whose geographic range is expanding due to climate change, and how they adapt to these changes and increase temperatures, considering global trade’s ability to exacerbate the spread of postharvest fungal contaminants of crops. Another important future direction is to fill the knowledge gap regarding the role of heat-resistant fungi (mainly belonging to the *Neosartorya* genus) in the enhancement of carbon sequestration, given the prospects and challenges of prolonging the postharvest shelf life of fruits, vegetables, crops, and food.

## Figures and Tables

**Table 1:** Synonymous *Neosartorya* spp. species.

Species Name	Synonymous Species	High Similarity or Identical Features	Status	Ref.
*Neosartorya otanii*	*N. fennelliae*	Identical β-tubulin gene sequences, no differences in ascospores	Mating experiments needed for proof	[21]
*N. spinosa*	*N. botucatensis*, *N. paulistensis*, *N. takaki*	Circular arrangements on the convex walls of ascospores	Accepted	[22]
*Neosartorya primulina*	*N. quadricincta*	Nearly identical gene sequences for β-tubulin, calmodulin and actin, morphology, ascospore ornamentation, restricted growth on Czapek agar	Accepted	[22]
*Neosartorya delicata*	*N. tatenoi*	Identical ascospore morphology, nearly identical gene sequences for β-tubulin, calmodulin and actin	Accepted	[22]

## Data Availability

Not applicable.
